# Optimization of ultrasonic-assisted enzymatic extraction of kiwi starch and evaluation of its structural, physicochemical, and functional characteristics

**DOI:** 10.1016/j.ultsonch.2021.105866

**Published:** 2021-12-08

**Authors:** Jiaqi Wang, Tian Lan, Yushan Lei, Jiangtao Suo, Qinyu Zhao, Haoli Wang, Jing Lei, Xiangyu Sun, Tingting Ma

**Affiliations:** a College of Food Science and Engineering, College of Enology, Northwest A&F University, Yangling 712100, China; b Shaanxi Rural Science and Technology Development Center, Xi’an 710054, China; c Shaanxi Bairui Kiwifruit Research Co, Ltd., Xi’an 710054, China

**Keywords:** Kiwi starch, Ultrasonic-assisted enzymatic extraction, Characteristics, Antioxidant capacity, Digestive resistance

## Abstract

A new ultrasound-assisted enzymatic extraction (UAEE) method of starch from kiwifruit was established and optimized using response surface methodology (RSM). Under optimal conditions (the pectinase-to-cellulase-to-papain ratio = 1:2:1 g/kg, solid/liquid ratio = 1:6.68, extraction pH = 5.23, ultrasound power = 300 W, and extraction temperature = 52 °C), the kiwi starch (KS) yield was about 4.25%, and the starch content of KS was 873.23 mg/g. Compared to other extraction methods, UAEE can obtain KS with high yield and purity with a shorter extraction time and less solvent and enzyme. The extracted KS has a low gelatinization enthalpy (8.02 J/g) and a high peak viscosity (7933 cP), with obvious particle properties and low adhesion. In addition, KS is rich in polyphenols, has strong antioxidant activity, and has higher contents of amylose starch (30.74%) and resistant starch (60.18%). This study established a novel and highly efficient method for KS extraction and suggest several possible applications for KS in the food industry.

## Highlights

• Superior kiwi starch (KS) was obtained by an ultrasonic-assisted enzymatic method.
• Research the structural, physicochemical, nutritional, and functional properties of KS.
• KS is rich in polyphenol (2543.52 μg GAE/g), exhibiting strong antioxidant capacity.
• KS is rich in RS (60.18%), indicating it has strong digestive resistance.

## Graphical abstract

**Figure:**
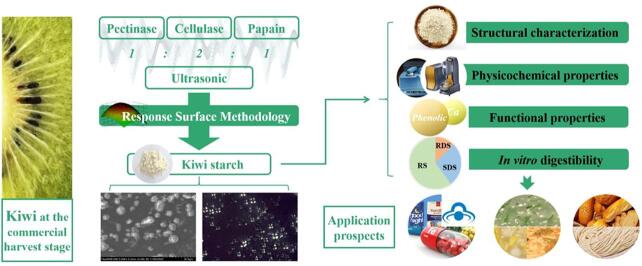

## 1 Introduction

Kiwifruit is considered one of the most commercially valuable fruits today because of its rich nutrition and sweet and sour taste [1]. During kiwifruit growth, the starch content (dry weight (DW)) gradually increases from 2% to about 40–60%. And starch is regarded as one of the main components of kiwifruit at the commercial harvest stage [2]. Due to natural shedding, diseases and insect pests, artificial thinning, and fruit grading, many kiwifruits that do not meet commercial standards are produced, and caused a huge waste. These fruits is rich in kiwifruit starch (KS), which is a source of starch that is not being foucsed [3]. Developing KS resources from these non-commercial fruit will provides a whole new way of disposal these resources, and significantly help building recycling kiwifruit agriculture, and improve the returns of kiwi growers.

KS is a type of fruit-derived starch. Generally, fruit-derived starches not only have the basic functional properties of certain traditional starches (such as corn starch and potato starch) but also have some unique nutrients and functional properties, such as a unique fruit flavor, rich in polyphenols, strong antioxidant activity, etc. [4], [5], [6]. For example, immature bananas are considered the best source of resistant starch in non-processed food [7], which can maintain physical health and establish a better intestinal microecological environment [8]. In Southeast Asian countries, canna starch has become a substitute for mung bean starch to make noodles due to the advantages of high transparency, low cooking loss, and light taste [9]. KS had been proved with a different property compared with traditional starch in some aspects. The pH value of KS is lower, and its dietary fiber content, free phenol content, and in vitro antioxidant capacity are significantly higher than those of potato, corn, and wheat starch, and KS has higher rheological properties. When added to traditional starch as a food supplement, KS can significantly improve the functional properties of native starch, and benefits health [10]. Therefore, KS is viewed as a type of health food that can be add value to processing.

However, at present, information about KS is scarce, and the extraction method is only limited to the traditional alkali extraction method, which is time-consuming, more impurities, and the purity is not ideal [3], [10]. This largely hinders the in-depth research, development, and utilization of KS. Different extraction processes significantly affect the purity, structural characteristics and biological activity of starch. Ultrasonic extraction and enzymatic extraction had been proved could significantly shorten the extraction time, reduce energy consumption, and increase the extraction rate, so are considered as green and efficient starch extraction methods [11]. The ultrasonic-assisted enzymatic method has been applied to other fruit starches, such as Arenga pinnata starch [12] and lotus seed starch [11]. However, no researchers have applied a combination of the two to kiwi starch. Thus, the aim of this study was to establish an innovative method for extracting starch from kiwifruit by UAEE. RSM was used to optimize the extraction parameters. The structural characteristics, and physicochemical, nutritional, and functional properties of the extracted KS were systematically studied. The in vitro digestion of KS was also evaluated. The findings are expected to provide a theoretical basis and technical support for the industrial production and application of KS.

## 2 Materials and methods

### 2.1 Materials and chemicals

Huayou kiwifruit was purchased from a local orchard in Mei County, Shanxi Province, China. Porcine pancreatic α-amylase (23 U/mg); pancreatic lipase (57 U/mg); invertase (≥300 U/mg); glucosidase (8 × USP); gallic acid; catechin 6-hydroxy-2,5,7,8-tetramethylchroman-2-carboxylic acid (Trolox); 1,1-diphenyl-2-picrylhydrazyl (DPPH); and Folin–Ciocalteu reagent were all purchased from Sigma-Aldrich Co. Ltd. (St. Louis, MO, USA). Pectinase (500 U/mg); cellulase (50 U/mg), papain (800 U/mg); 2,4,6-tripyridine-s-triazine (TPTZ); and potassium bromide (Spectrography) were purchased from Yuanye Biotechnology Co., Ltd. (Shanghai, China). The starch content test kit and glucose test kit (glucose oxidase method) were purchased from Nanjing Jiancheng Bioengineering Institute. Other reagents were analytical pure and were purchased from Xinfang Chemical Reagent Co., Ltd. (Yangling, China).

### 2.2 Extraction methods

Six batches of kiwis were picked during commercial harvest period, when their hardness were 90–100 N and the content of soluble solids were 6.5–8.0 Brix°. Kiwi fruit were washed, peeled, and removed the seeds. Adding ice water at a 20% mass of the fruit (FW) mass and used a high-speed blender to beat the pulp. Add a certain amount of pectinase, cellulase, and papain; adjusted the extraction pH of the system with 2% NaOH solution; and set the appropriate ultrasonic conditions. After enzymolysis, the mixed slurry was centrifuged at 11,520 × *g* for 10 min, the supernatant was poured out, and the precipitate was dissolved in 85% ethanol to wash off the glucose in the precipitation. The slurry was centrifuged twice for 10 min each. The precipitate, after alcohol washing, was re-dissolved in water and centrifuged again at 11,520 × *g* for 10 min; the supernatant was discarded; and a precipitate was obtained. Pave the precipitates, dry them in the oven at 30℃, grind them, and sieve them for 100 mesh to obtain KS. The flow chart of extraction was as Figure S1:

### 2.3 Contrast test of enzymatic hydrolysis strengthening combination

Take 100.0 g of kiwifruit homogenate (FW), and add the prepared hydrolase solution in a ratio of 5 times the weight of the fruit pulp. The hydrolase solution preparation method is shown in Figure S2. Adjust the extraction pH of the enzymatic hydrolysate to 5.0 with 2% NaOH and set the ultrasound power to 200 W, the extraction temperature to 50 ℃, and the ultrasound time to 60 min. Then, proceed to extract KS according to the steps in Section 2.2 and determined the starch content in the extract after sieving. Take the maximum starch content of the final extract as the index to determine the best combination of enzymatic hydrolysis.

### 2.4 Single factor experiment

Take 100.0 g of kiwifruit homogenate (FW) and add the prepared hydrolase solution with a certain liquid–solid ratio. For the preparation of the hydrolase solution, the pectinase-to-cellulase-to-papain ratio was 1:2:1 g/kg. Adjust the extraction pH of the enzymatic hydrolysate with 2% NaOH. The single factor test design and specific parameters are listed in Table S1. Then follow the step in Section 2.2 to extract KS and determine the starch content. The maximum starch content in the final extract was used as the index to determine the design level in the subsequent response surface experiment.

### 2.5 Box-Behnken design (BBD)

Through the single factor experiment, four factors were selected to optimize, including liquid/solid ratio, extraction pH, ultrasound power, and extraction temperature. The starch content of the final extract was chosen as the response value. The test factors and level design are shown in Table 1.

**Table 1:** Experimental design and results for response surface analysis.

Test group	Coded levels					Response value	
	A Liquid-solid ratio (mL/g)	B Extraction pH	C Ultrasound power (W)	D Extraction temperature (◦C)		Measured value (mg/g)	Predictive value (mg/g)
1	3(-1)	4.5(-1)	300(0)	50(0)		724.38	716.12
2	9(1)	4.5(-1)	300(0)	50(0)		629.43	643.27
3	3(-1)	5.5(1)	300(0)	50(0)		739.52	732.78
4	9(1)	5.5(1)	300(0)	50(0)		839.36	854.71
5	6(0)	5(0)	250(-1)	45(-1)		726.25	734.73
6	6(0)	5(0)	350(1)	45(-1)		744.43	744.26
7	6(0)	5(0)	250(-1)	55(1)		821.65	828.92
8	6(0)	5(0)	350(1)	55(1)		781.18	779.79
9	3(-1)	5(0)	300(0)	45(-1)		674.31	680.28
10	9(1)	5(0)	300(0)	45(-1)		737.85	754.76
11	3(-1)	5(0)	300(0)	55(1)		800.32	795.08
12	9(1)	5(0)	300(0)	55(1)		763.99	769.68
13	6(0)	4.5(-1)	250(-1)	50(0)		658.79	671.26
14	6(0)	5.5(1)	250(-1)	50(0)		855.39	865.93
15	6(0)	4.5(-1)	350(1)	50(0)		730.97	732.09
16	6(0)	5.5(1)	350(1)	50(0)		766.32	765.51
17	3(-1)	5(0)	250(-1)	50(0)		848.34	845.48
18	9(1)	5(0)	250(-1)	50(0)		715.23	679.34
19	3(-1)	5(0)	350(1)	50(0)		617.86	635.00
20	9(1)	5(0)	350(1)	50(0)		866.11	850.22
21	6(0)	4.5(-1)	300(0)	45(-1)		667.24	651.44
22	6(0)	5.5(1)	300(0)	45(-1)		811.36	795.97
23	6(0)	4.5(-1)	300(0)	55(1)		750.15	746.78
24	6(0)	5.5(1)	300(0)	55(1)		833.31	830.35
25	6(0)	5(0)	300(0)	50(0)		877.82	866.11
26	6(0)	5(0)	300(0)	50(0)		867.23	866.11
27	6(0)	5(0)	300(0)	50(0)		869.62	866.11
28	6(0)	5(0)	300(0)	50(0)		853.21	866.11
29	6(0)	5(0)	300(0)	50(0)		862.69	866.11

### 2.6 Structural characterization

#### 2.6.1 Scanning electron microscopy (SEM) and polarizing microscope (PLM)

Fix a small amount of starch powder (DW) on the metal sample table with conductive double-sided tape and observed it under a SEM (FlexSEM1000, Hitachi, Tokyo, Japan), at an accelerating voltage of 5.0 kV. A small amount of 2% (w/v) KS milk was placed on the sample stage of a PLM (XPV-230E, Shanghai Changfang Optical Instrument Co., Ltd., China) for observation and photography.

#### 2.6.2 Particle size distribution (PSD)

PSD was determined according to the method of Ding et al. [13] with minor modifications. A total of 100 mg KS (DW) was weighed, suspended in water, dispersed by ultrasonic wave, and then injected into the sample. An LS13320 laser particle size analyzer (Beckman Coulter, Inc., CA, USA) was used to automatically measure the size and distribution of starch particles. The data of sample size distribution were automatically processed and analyzed the analyzer’s own software.

#### 2.6.3 X-ray diffraction (XRD)

XRD was performed according to the method of Wang et al. [14] with minor modifications. The XRD test of KS was performed using a Bruker D8 Advance A25 X-ray diffractive analyzer (Germany) equipped with Cu-Kα radiation, where 2θ = 5-40° and step size 0.02°. The crystallinity was obtained using the Jade 6.5 software (Materials Data, Inc., Livermore, California, USA).

#### 2.6.4 Fourier transform infrared spectroscopy (FTIR)

FTIR was performed according to the method of Wang et al. [14] with minor modifications. The FTIR spectra of the samples were obtained by an FTIR spectrometer (Vetex70, Bruker, Germany). The resolution was 4 cm^−1^ and the spectral range was 4000–400 cm^−1^. The starch sample (DW) was mixed with potassium bromide and then pressed into tablets, where the ratio of sample to potassium bromide was 1:100. The number of replicates per samples was three, background corrections were performed and normalisation of spectra was performed.

### 2.7 Physicochemical properties

#### 2.7.1 pH and transparency

1.000 g KS (DW) was accurately weighed to prepare 1.0% starch milk. The pH of the starch milk was measured using a PHS-3E pH meter (Shanghai Leici Co. Ltd., Shanghai, China). The light transmittance of starch paste was determined following the method of Zhang et al. [15].

#### 2.7.2 Water soluble index (WSI) and swelling power (SP)

The WSI and SP were determined according to the method of Wang et al. [14], with a slight change. Put 500 mg (W_0_, on a dry basis) of KS into a plastic centrifuge tube, added 50 mL of distilled water, and heated it in a water bath at 85 ℃ for 30 min. Then, immediately cool the centrifuge tube to room temperature in an ice bath and centrifuged it at 3000 × *g* for 30 min. The supernatant was carefully poured out and dried to constant weight (W_1_) at 110 ℃, and the precipitate (W_s_) was accurately weighed. The WSI and SP of KS were calculated according to the following formulas:

(1) $$\mathrm{WSI}=(W_{1}/W_{0})\times 100\%$$

(2) $$\mathrm{SP}=W_{s}/\left(W_{0}\times \left(100\%\;-WSI\right)\right)\;\left(g/g\right)$$

#### 2.7.3 Differential scanning calorimetry (DSC)

The DSC was performed with a slightly modified method of Zhang et al. [16]. KS (3 mg, DW) was accurately weighed in a special liquid crucible, and then deionized water was added at a starch/deionized water ratio of 1:3. Seal the crucible and equilibrate at 4 ℃ for about 15 h. An empty crucible was used as a reference. DSC (Q2000, TA Instruments, USA) was used to determine the thermodynamic properties of KS during gelatinization according to the following procedures: keeping at 25 ℃ for 1 min, heating continuously to 115 ℃ at the rate of 10 ℃/min, and then decreasing to 40 ℃ at a rate of 10 ℃/min.

#### 2.7.4 Pasting properties

The pasting properties of starch were measured using a Rapid Visco-Analyzer (RVA-Tec Master, Perten Instruments, Sweden). The standard 1 program of the instrument was used for the measurement; except for the pre-shearing process (960 rpm), the blade rotation speed was 160 rpm. The temperature of the starch paste reached 50 ℃ in 1 min, was heated to 95 ℃ in 4 min and 42 s, maintained at 95 ℃ for 2 min and 30 s, then cooled to 50 ℃ in 11 min, and maintained for 2 min; at 13 min, the process was terminated. After that, the starch was gelatinous. Time to peak viscosity (Ptime), pasting temperature (PT), peak viscosity (PV), hot paste viscosity (HPV), cold paste viscosity (CPV), breakdown value (BD), and setback value (SB) were automatically analyzed by the analyzer’s software [14].

#### 2.7.5 Rheological properties

The gelatinized starch paste obtained in Section 2.7.4 was tested to determine its rheological properties. Rheometers (DHR-1, Waters, MA, USA) was used. A PP40 clamp was selected. The measure gap was 1000 μm and measure temperature was 25 ℃. The shear stress of KS was measured in the process of increasing (0.1 to 1000 s^−1^) and decreasing (1000 to 0.1 s^−1^) with shear rate. After shear scanning analysis, the variation in the storage modulus (G'), the loss modulus (G''), and the loos tangent (tan δ = G''/G') with angular frequency from low (0.1 Hz) to high (20 Hz) frequency were measured simultaneously at 25 ℃, and the strain remained unchanged [16].

20% starch milk (w/v) was prepared for temperature scanning. In the linear viscoelastic region of the sample (the strain value was set to 2%), the temperature was heated at a rate of 5 ℃/min. The changes in G', G“ and tan δ with temperature during the process from low temperature (25 ℃) to high temperature (90 ℃) and back to low temperature (25 ℃) were measured [16].

#### 2.7.6 Gel texture properties

The gel properties were determined following a slightly modified method of Li and Zhu [3]. Prepare 20% starch milk (w/v) and place it in a sealed glass bottle in a shaking water bath (160–190 rpm) at 95 °C. The sample was stored at 4 °C for 24 h prior to testing. A TA-XT Plus Texture Analyzer (Stable Micro Systems Ltd., Godalming, UK) was used to study the gel texture properties in Texture Profile Analysis (TPA) mode. The test parameters were as follows: the P/0.5R probe was used, the test distance was 5 mm, the probe speed was 1 mm/s in the whole test process, the force sensing was 5 g, and the interval between adjacent tests was 5 s.

### 2.8 Functional properties

#### 2.8.1 Nutritive composition determination (starch content, amylose content, moisture content, TPC, and element content)

Starch content in KS was tested using a starch content test kit (A148-1–1 Starch). The content of amylose was determined following the method of Lan et al. [17]. The moisture content of KS was determined according to the first method of GB 5009.3–2016. The total polyphenol content (TPC) was determined by Folin-Ciocalteu colorimetry [1], the results are expressed as μg gallic acid equivalents (GAE)/g (μg GAE/g). The elemental content was determined following the method reported by Zhang et al. [1].

#### 2.8.2 Antioxidant activities (FRAP and DPPH)

The antioxidant capacity of the KS was determined by the DPPH and FRAP methods based on previous reports [18]; the results are expressed as μmol trolox equivalents per g (μM TE/g).

### 2.9 *In vitro* digestibility

In vitro digestion was determined following the method reported by Li and Zhu [3] with a few modifications. Disperse the starch sample (1.200 g, DW) in ultrapure water (8.00 mL) and stir continuously for 30 min in a boiling water bath to obtain gelatinized starch. 200 mg pancreatic lipase and 6 mL ultrapure water were stirred for 10 min to obtain the supernatant. Then, 2.5 mg invertase, 1 mL ultrapure water, and 1.5 mL supernatant were mixed with 10 μL glucosidase and 1 mL ultrapure water to obtain the enzyme solution. Mix the gelatinized starch cooled to room temperature with 10 mL of pH 5.2 sodium acetate buffer and the enzyme solution in an oscillating water bath at 37 ℃ (160–190 rpm). Samples were obtained at 0, 20, 40, 60, 80, 100, 120, 180, and 300 min. At each time point, 0.5 mL samples were placed into a centrifuge tube containing 20 mL of 75% ethanol solution, centrifuged at 8952 × *g*/min for 5 min, and the supernatant was removed to determine the glucose content.

### 2.10 Statistical analysis

The experimental results of the response surface design were analyzed using Design-Expert 8.0.6.1 software (State-Ease Inc., Minneapolis, MN, USA). One-way analysis of variance (ANOVA) and Duncan’s multivariate test were performed in SPSS 23 (SPSS Inc., IBM) (*p* < 0.05). RStudio-1.1.463 (RStudio, Inc., USA) was used for the correlation test. Excel 2016 (Microsoft, USA) and Origin 9.1 (OriginLab, USA) were used for other image rendering. All the experiments were performed at least in triplicate. The data are expressed as the mean ± standard deviation (SD).

## 3 Results and discussion

### 3.1 Enzyme type and strengthening method optimization

The effect of enzyme type and combinations on the KS content is shown in Figure S2. The synergism of pectinase, cellulase, and papain in the starch extraction was better than the combination of pectinase and cellulase. The extraction effect of the pectinase-to-cellulase-to-papain ratio at 2:1:1 or 1:2:1 was significantly better than that of the 1:1:2 pectinase-to-cellulase-to-papain ratio. The reason for this finding may be that kiwifruit is rich in pectin, cellulose, and protein. The structure of the kiwifruit cell wall could not be fully destroyed when certain enzymes were lacking, and it was difficult to effectively enzymatically decompose related components. Different types of compound enzymes in the appropriate proportion may stimulate enzyme synergy, which can damage the cell wall, increase the permeability of the cell membrane, and effectively improve the effect of enzymatic hydrolysis [19]. The extraction effect of combination 3 was slightly higher than that of combination 2, but there was no significant difference between the two combinations (*p* > 0.05). In conclusion, combination 3, the pectinase/cellulase/papain ratio of 1:2:1 was selected as the best combination for extracting KS.

### 3.2 Single factor experimental analysis

#### 3.2.1 Effect of liquid/solid ratio on starch content

The effect of different liquid/solid ratios (1, 3, 5, 10, and 15 mL/g) on starch content is shown in Figure S3A. When the liquid/solid ratio was 1 mL/g, the starch content of KS was lower (653.41 ± 10.19 mg/g); with the increase in the liquid/solid ratio, the starch content increased sharply and reached a maximum (795.22 ± 1.83 mg/g) at 5 mL/g. Then, it gradually decreased with the increase in liquid/solid ratio, which agrees with the findings of Zhang et al. [12]. This may be due to the significant decrease in the enzyme concentration when the liquid/material ratio is too high, which affects the enzymatic hydrolysis reaction.

#### 3.2.2 Effect of extract pH on starch content

The effect of different extraction pH values (4.0, 4.5, 5.0, 5.5, and 6.0) on starch content is shown in Figure S3B. The KS content first increased and then decreased with the increase in pH, potentially because the optimal pH values of the three enzymes used in this experiment are different [20], [21]. When the pH was 5.0, the comprehensive activity of the three enzymes was the highest, the reaction was the most thorough, and the impurities removal effect was the best. Under these conditions, the obtained KS content was the highest (814.92 ± 4.65 mg/g).

#### 3.2.3 Effect of ultrasound power on starch content

The effect of different ultrasound power (200, 250, 300, 350, and 400 W) on starch content is shown in Figure S3C. With the increase in ultrasound power, the starch content of KS first increased and then decreased, reaching a maximum when the ultrasound power was 300 W. In the early stages, with the increase in ultrasound power, the fluctuation of the sound field was enough to produce violent vibration and cavitation collapse to prompt the rapid swelling of the kiwifruit cell wall. Higher ultrasound power ensured a sufficient cavitation range, the overall mass transfer resistance was effectively reduced, and the extracted KS content increased [22]. However, when the power was too high, a large amount of hydroxyl radicals might be generated, leading to the chemical decomposition of starch [23], resulting in the decrease in starch content. Therefore, 300 W was considered to be the optimal extraction power in this experiment.

#### 3.2.4 Effect of extraction temperature on starch content

The effect of different extraction temperatures (35, 40, 45, 50, and 55 ℃) on starch content is shown in Figure S3D. The starch content reached a maximum at 50 ℃. This might be because, at a low temperature, increases in temperature lead to the intensification of molecular motion, thus accelerating the dissolution rate of small molecular compounds in kiwifruit cells. The appropriate temperature allows complex enzymes fully to play their role in enzymolysis [24]. Once the optimal temperature of the enzyme was exceeded, the structure of the enzyme would be damaged, significantly reducing the enzyme activity, resulting in a sharp decline in KS content.

#### 3.2.5 Effect of ultrasound time on starch content

The effect of different ultrasound time (30, 60, 90, 120, and 150 min) on starch content is shown in Figure S3E. Within a certain time range (30–60 min), the starch content gradually increased with ultrasound time, but did not change significantly between 60 and 120 min, and decreased significantly between 120 and 150 min. The reason for this finding might be that when the ultrasound time is too long, the strong ultrasonic cavitation and thermal effects degrade or denature the starch in kiwifruit , which leads to a decrease in starch content [25]. From the perspective of energy savings and practical production, the ultrasound time should be 60 min.

### 3.3 Optimization of extraction conditions

According to the design principle of the BBD test and the results of the single-factor test, four factors were selected: liquid/solid ratio, extraction pH, ultrasound power, and extraction temperature. The extraction process of KS was optimized based on the single-factor test using a four-factor, three-level response surface analysis method. The results of the single-factor tests showed that the starch content of each single-factor group reached the highest when the solid/liquid ratio was 1:5, the extraction pH was 5.0, the ultrasound power was 300 W, and the extraction temperature was 50 ℃. The RSM experimental results of ultrasonic-assisted enzymatic hydrolysis to extract KS are shown in Table 1.

Using Design-Expert 8.0.6.1 and the test data in Table 1, the quadratic polynomial regression equation of the liquid–solid ratio (A), extraction pH (B), ultrasound power (C), and extraction temperature (D) on starch content was obtained after regression fitting:

(3) $$\text{Y}=-67.792\;{\text{A}}^{2}-61.602\;{\text{B}}^{2}-45.8145\;{\text{C}}^{2}-48.3745\;{\text{D}}^{2}+48.6975\;\text{AB}+95.34\;\text{AC}-24.9675\;\text{CE}-40.3125\;\text{BCE}-15.24\;\text{BD}-14.6625\;\text{CD}+12.27\;\text{A}+57.025\;\text{B}-9.898333333\text{C}+32.43\;\text{D}+866.114$$

where Y is the predicted value of starch content; and A, B, C, and D are the coding value of the independent variables as described above.

The results of the variance analysis of the response surface model and the significance test of the model coefficients are shown in Table 2. From Table 2, the model established in this experiment was extremely significant (*p* < 0.01), and the lack of fit was not significant (*p* > 0.05), indicating that the model was appropriate. The coefficient of determination (R^2^) was 0.9529, indicating that the model explained about 95.3% of the response value changes. The correlation coefficient of the model (R) was 0.9764, indicating that its fitting degree was good and the experimental error was small. So, the KS extraction process could be analyzed and predicted.

**Table 2:** ANOVA for the response surface quadratic model of KS.

Source	Sum of Squares	df	Mean Square	F Value	p-value Prob > F	
Model	165623.9	14	11830.28	41.45356	< 0.0001	significant
A Liquid-solid ratio	1806.635	1	1806.635	6.330487	0.0247	
B Extraction pH	39022.21	1	39022.21	136.7346	< 0.0001	
C Ultrasound power	1175.724	1	1175.724	4.119762	0.0618	
D Extraction temperature	12620.46	1	12620.46	44.22235	< 0.0001	
AB	9485.786	1	9485.786	33.23839	< 0.0001	
AC	36358.86	1	36358.86	127.4022	< 0.0001	
AD	2493.504	1	2493.504	8.737292	0.0104	
BC	6500.391	1	6500.391	22.77751	0.0003	
BD	929.0304	1	929.0304	3.255342	0.0927	
CD	859.9556	1	859.9556	3.013303	0.1045	
A^2	29810.3	1	29810.3	104.4559	< 0.0001	
B^2	24614.96	1	24614.96	86.25134	< 0.0001	
C^2	13614.93	1	13614.93	47.707	< 0.0001	
D^2	15178.98	1	15178.98	53.18746	< 0.0001	
Residual	3995.41	14	285.3864			
Lack of Fit	3666.605	10	366.6605	4.460523	0.0813	not significant
Pure Error	328.8049	4	82.20123			
Cor Total	169619.4	28				
R-Squared = 0.9764, Adj R-Squared = 0.9529, C.V. % = 2.1837						

From Table 2, the quadratic term influence of the model in the regression equation was extremely significant (*p* < 0.01); the interaction between the factors and the primary term had both significant and insignificant effects. According to the F value, the main effect sequence of the four influencing factors could be judged as follows: extraction pH > extraction temperature > liquid/solid ratio > ultrasound power.

Figure S4 depicts a 3D response surface diagram providing a graphical interpretation of the regression equation and intuitively reflecting the influence of various factors on KS content. Figure S4A-F shows the relationship between the extraction variables and the KS content obtained by UAEE. Liquid/solid ratio, extraction pH, and extraction temperature had significant effects on KS content. With the increase in these three variables within a certain range, starch content increased significantly, and when these variables exceeded a certain value, they showed a stable trend or gradually decreased. In addition, it could be found that ultrasound power has little effect on KS content in UAEE, which is consistent with the conclusion in Table 2.

### 3.4 Verification of the predictive model

After optimization by RSM, the obtained optimal UAEE conditions for KS content were: solid/liquid ratio of 1:6.68, extraction pH of 5.23, ultrasound power of 292.43 W, and extraction temperature of 51.66 °C. Under these conditions, the theoretical yield of KS is 885.63 mg/g. To further verify the results, the above conditions were used for verification experiments. Considering the convenience of actual operation, the extraction conditions were optimized as follows: solid/liquid ratio of 1:6.68, extraction pH of 5.23, ultrasound power of 300 W, and extraction temperature of 52 °C. From the results of three repeated experiments was that the KS content was 873.23 ± 8.39 mg/g, and the relative error between the measured value and the theoretical prediction was only 0.84%; the yield of KS was about 4.25%. Therefore, the optimized UAEE process parameters based on RSM are accurate and reliable, and have application value.

### 3.5 Structural characterization

**Fig. 1:**
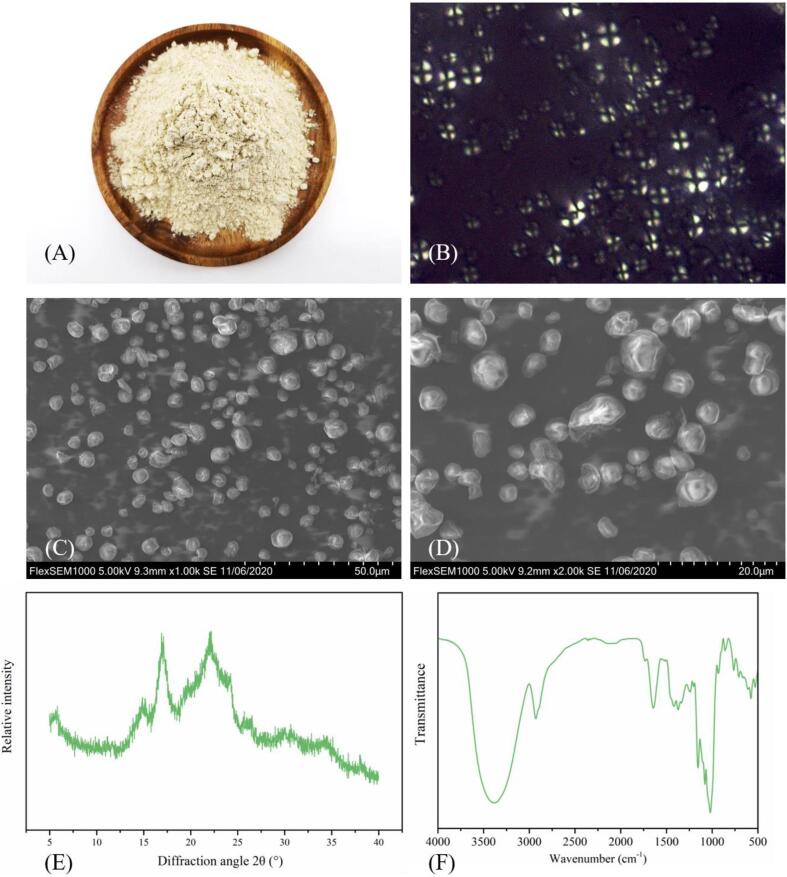
Structural characterization of KS. (A) Sample pictures; (B) PLM images of KS; (C) SEM images of KS (1000 × ); (D) SEM images of KS (2000 × ); (E) XRD and (F) FTIR spectra of KS.

The KS sample extracted by UAEE is shown in Fig. 1A. It appears as a milky white to light yellow powder with a particle size of about 8.33 μm (Table 3). As shown in Fig. 1B, the shape of KS was irregular, a clear polarized cross-structure was observed, and the crosses were basically located in the center of the particles. SEM images provided visual evidence of sample structure. As shown in Fig. 1C-D, KS particles had different sizes and similar irregular ellipsoid shapes, which are obviously different from those of corn starch [26], cassava starch [27], and potato starch [28]. The surface of the starch particles was relatively complete and smooth, with some small cracks in some particles.

**Table 3:** Structural, physicochemical, and functional characteristics of KS.

		Huayou
pH		5.32 ± 0.012
Transmittance (%)		1.27 ± 0.060
	
WSI (%)		29.63 ± 1.283
SP (g/g)		26.26 ± 0.394
Particle size (μm)		8.33 ± 0.128
Relative crystallinity (%)		48.62 ± 3.45
Thermal properties	To (℃)	63.71 ± 0.053
	Tp (℃)	67.90 ± 0.104
	Tc (℃)	74.15 ± 0.265
	ΔH (J/g)	8.02 ± 0.389
Pasting properties	PV (cP)	7933 ± 33.511
	HPV (cP)	2207 ± 7.550
	BD (cP)	5728 ± 21.633
	CPV (cP)	5717 ± 16.000
	SB (cP)	3501 ± 17.502
	Ptime (min)	4.92 ± 0.021
	PT (℃)	72.58 ± 0.076
Gelatinization properties	Hardness (g)	411.85 ± 10.091
	Springiness	0.92 ± 0.072
	Cohesiveness	0.32 ± 0.010
	Gumminess (g)	159.05 ± 4.540
	Chewiness (g)	138.41 ± 4.633
	Resilience	0.06 ± 0.004
	Adhesiveness	−48.691 ± 1.628
Starch content (mg/g)	873.23 ± 8.39	
Amylose content (%)	30.74 ± 0.023	
Moisture content (g/100 g)	11.08 ± 0.003	
Total polyphenol (μg GAE/g)	2543.52 ± 24.50	
Element content (mg/kg)	Cu	N.A.
	Zn	3.00 ± 0.10
	Na	1777.00 ± 55.25
	K	617.23 ± 15.46
	Ca	973.23 ± 55.98
	Mg	1830.25 ± 10.99
	Mn	24.20 ± 1.22
	Fe	78.11 ± 2.10
	P	363.26 ± 17.23
Antioxidant Activity (μM TE/g)	FRAP	29.17 ± 0.444
	DPPH	1.93 ± 0.047

The XRD of KS particles (Fig. 1E) showed almost no impurity peaks, confirming that the sample was mainly composed of hydrocarbons [29]. The sample had several distinct characteristic peaks in the range of 5°-40°, which were located at 5.6°, 14.8°, 17.0°, 22.2°, and 24.2°. However, the intensity of the diffraction peaks at 15.1°, 19.7°, and 34.5° was weak and the peak shape was not obvious. This result showed that the KS granules were a mixture of crystalline and amorphous, with an incomplete crystal structure, and presented the typical XRD spectrum characteristic of B-type crystalline starch. For example, banana starch often exhibits these characteristic peaks [5]. The diffraction peak near 2θ = 20° could be attributed to the amorphous peak of amylose and lipid [30]. This peak exists in common crops such as rice starch, potato starch, and pea starch, but the peak intensity varies widely because the lipid content in different starches is different [6]. The crystallinity of KS was low (48.62%), so the XRD diffraction peak intensity was low and the position was not obvious.

FTIR spectroscopy is commonly used to identify functional groups of substances, and to characterize the short-range ordered structure of starch [14]. As shown in Fig. 1F, the wavelength of 2937 is the polymerization bond absorption peak of alcohol or phenol, the wavelength of 1641 is the alkene bond absorption peak of α, β-unsaturated ketone, the wavelength of 1424 is the carbonyl acid absorption peak, and the wavelength of 1163 is the peak of carbonyl ester, which preliminarily indicated that KS contains some nutrients that were not found in traditional starch, such as phenols and ketones, which might contribute to its unique antioxidant properties. The crystalline region of starch is formed by the transverse arrangement of double-helix branched chains of amylopectin into a lattice. The short-range arrangement of helices is called the double-helix arrangement [4]. The degree of order of starch can be quantified by amplitude ratio 1047/1022 cm^−1^ (R_1047/1022_) [14]. The R_1047/1022_ of KS was 1.18, similar to that sweet potato starch (1.05) [14], but significantly higher than that of jackfruit (0.610), longan (0.576), litchi (0.606), loquat (0.618), mango kernel starch (0.654) [4], and wheat starch (0.867) [31]. Overall, the short-range structure of KS has a higher degree of order.

### 3.6 Physicochemical properties

#### 3.6.1 pH, transparency, SP and WSI

The pH of KS extracted by UAEE is 5.32 (Table 3), which is weakly acidic, affecting the processing characteristics of KS. As shown in Table 3, the transparency of KS is only 1.27%, which is much lower than that of traditional starches such as wheat starch [32] and corn starch [33]. So, KS might be more suitable for foods with low transparency requirements such as soups and sauces. The transparency of KS largely depends on the amylose content, swelling capacity, and the remaining level of unexpanded particles [34]. As shown in Table 3, both the amylose content (30.74%) and SP (26.26 g/g) of KS are higher, which may directly lead to the low transparency of KS paste.

The study of the properties of starch–water systems in the processing of starch-based foods is important, as the WSI and SP of starch reflect the interaction between starch and water. Table 3 shows that after heating in a water bath at 85 °C for 30 min, the WSI of KS was 29.63 g/g and the swelling power was 26.26 g/g, which are significantly higher than those of banana starch (WSI = 16.8 g/g, SP − 17.1 g/g), which is also a fruit-derived starch [35]. The WSI and SP of starch are the result of the comprehensive action of starch particle diameter, gelatinization temperature, amylopectin chain length, amylose content, lipid content, and protein content [6].

#### 3.6.2 Differential scanning calorimetry (DSC)

The onset temperature (To), peak temperature (Tp), conclusion temperature (Tc), and enthalpy change (ΔH) were recorded by DSC, which were 63.71 ℃, 67.90 ℃, 74.15 ℃, and 8.02 J/g, respectively (Table 3). Compared to potato starch, there was no significant difference among To, Tp, and Tc, but ΔH was significantly reduced [28]. The ΔH reflects the energy required to dissociate the amylopectin double helix in crystal lamella, which is positively correlated with starch crystallinity [36]. The gelatinization enthalpy of KS is smaller than that of traditional starch, indicating that the gelatinization requires less energy to absorb and is easier to cook.

#### 3.6.3 Pasting properties

By studying the pasting properties of KS, its cooking performance and food utilization characteristics were predicted. After heating about 10% (w/w) starch milk according to the set procedure, the pasting properties of KS were obtained by RVA, as shown in Table 3. The PV of KS is about 7933 cP, which is even higher than that of potato starch [28]. The results indicated that the viscosity of KS paste extracted by UAEE is higher. The difference between the PV and the HPV is thought to be due to the breakdown of starch granules, and this difference is known as the BD. The HPV of KS is 2207 cP, and its BD is 5728 cP. The higher the BD of the sample, the lower the ability of the starch to withstand heating and shear stress during cooking. The CPV of KS is about 5717 cP, much higher than that of jackfruit starch (3001–4230 cP), which might be related to the high content of amylose in KS [37]. Due to its high viscosity, KS may be used as a thickener for foods. The SB represents the regenerative trend of starch; the SB of KS is relatively high (3501 cP), so it can be used as a filler in frozen products. In addition, the PT of KS is 72.58 ℃, and the Ptime is 4.92 min, which are significantly lower than that of *Malaysia 1* jackfruit starch (91.29 ℃ and 6.49 min, respectively), indicating that it was relatively easier to cook. Studies showed that the PT depends on the amylose content and the size of starch particles [37].

#### 3.6.4 Rheological properties

**Fig. 2:**
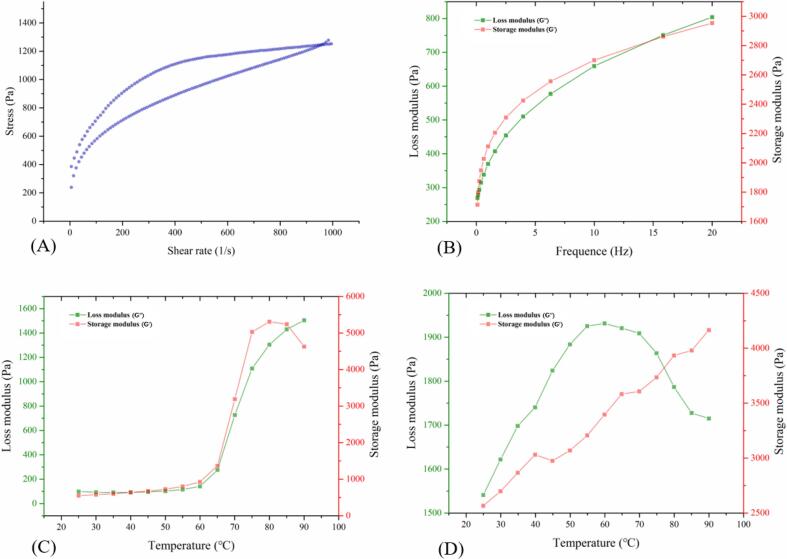
Rheological properties of KS. (A) The hysteresis loops of KS; (B) Changes in storage storage and loss modulus of KS suspensions during a frequency sweep (C) Changes in storage and loss modulus of 20% KS starch suspensions during heating; (D) Changes in storage storage and loss modulus of 20% KS suspensions during cooling.

KS paste can be regarded as a function of shear rate under shear stress (Fig. 2A). It is a pseudoplastic fluid with shear thinning behavior and can be well-described by the power law equation, and its determination coefficient (R^2^) is above 0.95 (Table S2). The difference in flow behavior of KS paste during the two shear processes indicated that the starch gel network undergoes irreversible changes under the action of shear force [10].

The dynamic rheology test was mainly used to determine the viscoelasticity of KS. Its main parameters are the storage modulus (G', characterizing elasticity), the loss modulus (G'', characterizing viscosity), and the loos tangent (tan δ, characterizes the damping capacity). As shown in Fig. 2B, in general, G'>G'', and both show an upward trend as the frequency increases. The KS has both liquid fluidity and viscosity as well as solid elasticity, and the elasticity is dominant. It was similar to the conclusion made by Wang et al. [14] in sweet potato starch. During the heating process (Fig. 2C), within the range of 25–60 ℃, G' and G“ were basically stable. After 60 ℃, G' and G” increased sharply and reached the maximum. As the temperature continued to rise, G' began to show a downward trend, and the growth rate of G“ slowed. When the temperature began to increase, the starch granules absorbed water and expanded, and amylose exuded from the starch granules and intertwined with each other to form a network structure [10]. However, further heating led to the melting, breaking, and disintegration of starch grains, and the weakening of interchain interactions, so G 'and G'' showed a different trend than before. During the cooling process (Fig. 2D), G' was always greater than G”, which indicated that elasticity still played a dominant role, and the system presented more solid characteristics.

#### 3.6.5 Gel texture properties

In TPA mode, the first pressure was applied to the sample to deform it to correspond to the first bite of human chewing, and then the second pressure was applied to determine the gel texture properties of starch by simulating human chewing movements. The results of TPA measurement are shown in Table 3. The hardness of 20% KS gel is 411.85 g, springiness is 0.92, cohesiveness is 0.32, gumminess is 159.05 g, chewiness is 138.41 g, resilience is 0.06, and adhesiveness is −48.691. Compared to potato starch and corn starch, KS has higher hardness and gumminess [38]. However, compared to sweet potato starch and quinoa starch, KS has higher adhesion and lower cohesiveness [39]. All these findings indicated that KS has strong granular properties, but low adhesion. Significant differences in TPA between KS and other starches might be related to amylose content, starch purity, and the different structures of amylose and amylopectin [37].

### 3.7 Functional properties

The purity of the KS extracted by the UAEE process is high. As shown in Table 3, its moisture content is 11.08 g/100 g, which meets the requirements of GB31637-2016 for starch (≤18 g/100 g). The amylose content of KS is 30.74%, much higher than previously reported for corn starch (17.1%) [38], tapioca starch (18.10%) [27], potato starch (25.6%), sweet potato starch (20.1%), and quinoa starch (17.7%) [39]. Its high amylose starches determine the physical, chemical, functional, and digestive properties of KS to a certain extent.

The kinds and contents of elements in KS were detected. Except for Cu, the other eight elements were detected in KS (Table 3). Among the macro elements, their amounts in decreasing order are: Na (1777 mg/kg) > Ca (973.23 mg/kg) > K (617.23 mg/kg) > P (363.26 mg/kg). Among them, P is an important element in starch. Ca is an important nutrient in kiwifruit, and the content of Ca in KS is much higher than in other starches [39]. Therefore, KS can be used as a good calcium supplement. Among the trace elements, the amounts in decreasing order are: Mg (1830.25 mg/kg) > Fe (78.11 mg/kg) > Mn (24.20 mg/kg) > Zn (3.00 mg/kg) > Cu (N.A.).

In addition to the nutrients contained in traditional starch, KS also retains some unique nutritional and functional characteristics of kiwifruit. For example, KS contains extremely high amounts of phenolic substances (2543.52 μg GAE/g). These phenolic compounds might be tightly bound to starch molecules, existing in a tightly bound state and surviving under high-intensity ultrasound. Zhang et al. [1] found that the content of phenolic compounds in kiwifruit is significantly and positively correlated with its antioxidant capacity. The results in Table 3 show that the sample exhibited strong iron atom reduction ability (29.17 μM TE/g) and DPPH radical scavenging activity (1.93 μM TE/g). This is an important characteristic that distinguishes KS from traditional starch.

### 3.8 In vitro digestibility

Starch is a staple food in people’s daily lives, and its digestion rate seriously affects the postprandial blood glucose level. According to the digestion behavior of starch under the action of enzymes, starch can be divided into three types: rapidly digestible starch (RDS), slowly digestible starch (SDS), and resistant starch (RS) [40]. SDS is considered to be the most ideal form of dietary starch [41], whereas RS is thought to provide health benefits, e.g., regulating blood glucose levels, promoting colon health, and inhibiting fat accumulation [7]. As shown in Figure S5B, RS has the highest content in KS (60.18%), followed by SDS (27.54%), then RDS (12.28%). KS is rich in RS, being significantly higher than in traditional starches such as corn starch (24.50%), potato starch (28.60%), and pea starch (37.34%) [42], and is close to that of banana starch, which is recognized as a natural source of resistant starch, indicating it is more difficult for enzymes to digest. This might be related to the rich phenolic substances. Studies showed that phenols can inhibit the activity of α-amylase and α-glucosidase and limit the hydrolysis of starch [43]. The digestion characteristics are also closely related to the particle size and amylose content of starch [6]. In the future, KS can be developed as a new source of resistant starch, or as an ingredient with potential physiological benefits in the food industry.

The digestion process of KS is shown in Figure S5A. However, KS did not reach the hydrolysis equilibrium within 300 min of the experiment. So, the digestion kinetics could not be analyzed. In subsequent experiments, if the digestive properties need to be further quantified, a longer digestion time may be needed to fully digest KS.

## 4 Conclusions

KS with high yield (4.25%) and purity (87.32%) was developed by the ultrasonic-assisted enzymatic extraction of KS by RSM for the first time. The starch particles obtained by this method are irregular in shape, with many edges and corners, showing a B-type crystal structure, with low relative crystallinity (48.62%) and high short-range structure order degree. The PV, BD, and SB of KS are all high, the elastic characteristics and particle properties are more obvious, and the gelatinization enthalpy is lower. Compared to other traditional starches, KS is rich in calcium (973.23 mg/kg), phenolic substances (2543.52 μg GAE/g), and KS (60.18%), exhibits strong antioxidant capacity and digestive resistance. The role of functional food formula of KS will be further researched.

### CRediT authorship contribution statement

**Jiaqi Wang:** Conceptualization, Methodology, Software, Investigation, Data curation, Writing – original draft. **Tian Lan:** Data curation, Writing – original draft. **Yushan Lei:** Visualization, Investigation, Supervision. **Jiangtao Suo:** Writing – review & editing. **Qinyu Zhao:** Software, Validation. **Haoli Wang:** Software, Validation. **Jing Lei:** Writing – review & editing. **Xiangyu Sun:** Writing – review & editing. **Tingting Ma:** Writing – review & editing.

## Declaration of Competing Interest

The authors declare that they have no known competing financial interests or personal relationships that could have appeared to influence the work reported in this paper.
